# Smoking behavior and circulating vitamin D levels in adults: A meta‐analysis

**DOI:** 10.1002/fsn3.2488

**Published:** 2021-08-05

**Authors:** Lu Yang, Hang Zhao, Ke Liu, Yichao Wang, Qianqian Liu, Tiantian Sun, Shuchun Chen, Luping Ren

**Affiliations:** ^1^ Hebei General Hospital Shijiazhuang China

**Keywords:** meta‐analysis, smoking, systematic review, vitamin D

## Abstract

To determine the effect of smoking on circulating vitamin D in adults, we performed a meta‐analysis. Literature before 9 May 2021 was retrieved from electronic literature databases such as EMBASE, PubMed, and Cochrane. The quality of the included studies was assessed by two researchers against the Newcastle–Ottawa scale and JBI Evidence‐based Health Care Centre criteria. All eligible studies and statistical analyses were performed using STATA 14. Twenty‐four studies with 11,340 participants meeting the criteria were included in the meta‐analysis. The results of meta‐analysis showed that the level of circulating 25(OH)D in smokers was lower than that in nonsmokers. A subgroup analysis based on vitamin D supplement use showed that both smokers who used vitamin D supplements and smokers who did not use vitamin D supplements had lower blood 25(OH)D levels compared with the control group. In addition, subjects were divided into different subgroups according to age for meta‐analysis, and the results showed that the serum 25(OH)D level in each subgroup of smokers was lower than that in the control group. This meta‐analysis revealed differences in circulating vitamin D levels between smokers and nonsmokers, with smokers likely to have lower circulating vitamin D levels.

## Graphical abstract

So far, there is no consensus on the effect of smokers on circulating vitamin D levels. This is the first meta‐analysis of circulating vitamin D levels in adult smokers. We found that smoking negatively affects circulating vitamin D levels in adults.

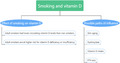

## 1 INTRODUCTION

It is well known that smoking is an important cardiovascular and cerebrovascular risk factor. Now, some studies have proved that smoking is not only a major harm to cardiovascular and cerebrovascular health, but also a major cause of chronic obstructive pulmonary disease. Smoking is associated with accelerated decline in lung function, increased mortality, and worsening symptoms in asthma and COPD (Boulet et al., 2006). There are also meta‐analyses that show smokers have an increased risk of fracture (Kanis et al., 2005; Shen et al., 2015).

Vitamin D is one of the most important fat‐soluble vitamins in the body, the most important of which are D2 and D3. As the main circulating form of vitamin D, 25(OH)D plays an important role in regulating cell proliferation and immune response, in addition to regulating serum calcium and phosphorus levels (Umar et al., 2018; Vanherwegen et al., 2017). Studies have found that inhibiting the production of cAMP protein induced by 1,25(OH) 2D3 can enhance the growth of mycobacterium (Liu et al., 2007). A meta‐analysis found that vitamin D deficiency increases the risk of ischemic stroke (Zhou et al., 2018). Vitamin D deficiency has also been related to type 2 diabetes (Berridge, 2017; Lucato et al., 2017) and may increase the risk of falls (Girgis et al., 2013). Vitamin D deficiency is now a major health problem in the real world.

Many studies have shown the effect of smoking on circulating 25‐hydroxyvitamin D, although someone has considered that no statistically significant effect on circulating vitamin D levels (Stürmer et al., 2015). However, there is growing evidence of the negative effects of smoking on 25‐hydroxyvitamin D and calcium metabolism (Cuomo et al., 2019; Cutillas‐Marco et al., 2012; Hermann et al., 2000; Kim et al., 2018; Richard et al., 2017). A study by Jääskeläinen et al. (2013) of 5,714 subjects (47% males) aged 30–79 years found that smokers had lower serum 25(OH)D concentrations than nonsmokers. Hermann et al. (2000) found that current smokers had significantly lower serum 25‐OHD levels than nonsmokers. Although the decrease was only 6.8%, serum levels were negatively correlated with the number of cigarettes currently smoked per day (*β* = −.16; *p* = .003). In addition, Thuesen et al. (2012) in a recent large population study showed that the odds ratios of severe vitamin D deficiency(25(OH)D < 10 ng/ml)/vitamin D deficiency(25(OH)D < 20 ng/ml) associated with daily smoking were 1.47 and 1.36, respectively. This cannot be explained by other confounding lifestyle factors. After adjustment for other confounders, the adverse effects of smoking on circulating vitamin D levels were still suggested (Brot et al., 1999).

There is no consensus on whether smoking causes damage to circulating vitamin D levels, which may have to do with differences in study design or participant characteristics (such as age, sex, weight, health status), and techniques (testing methods) between studies. In recent years, research has continued on whether low levels of vitamin D are common in smokers. To determine the impact of smoking behavior on circulating vitamin D levels, we conducted a meta‐analysis of published studies to draw conclusions and provide public advice on clinical and public health issues.

## 2 MATERIALS AND METHODS

### 2.1 Search strategy

Studies on smoking and circulating vitamin D published before 31 December 2020 were searched from the following databases: MEDLINE(PubMed), EMBASE, and Cochrane. The retrieval strategy was ((25‐hydroxyvitamin D[ALL] OR 25(OH)D[ALL] OR bone marker[ALL] OR Parathyroid Hormone[ALL] OR PTH[ALL]) AND (smoking[ALL] OR cigarette[ALL] OR smoker[ALL])).

### 2.2 Selection criteria

All studies included in this meta‐analysis met the following five criteria: (1) Diseases affecting vitamin D absorption or metabolism were excluded, (2) clear smoking status and circulating vitamin D data, (3) all subjects are 18 years or older, (4) reported the types of samples, and (5) compare the circulating vitamin D status of smokers with that of nonsmokers. Any of the following studies were excluded: participating in osteoporosis studies or not excluding diseases that affect vitamin D levels; data cannot be obtained, extracted, or documented (or if the author does not respond when attempting to contact); studies on minors; patients who use marijuana or drugs; and studies on vitamin D deficiency are ill‐defined.

### 2.3 Data extraction

The following information and clinical characteristics of the participants were extracted from the included study: first author, year of publication, research type, detection method, sample type, study location, average age, body mass index (BMI), number of participants, number of smokers and nonsmokers, circulating vitamin D levels in smokers and nonsmokers (dichotomous variables studies were used to investigate vitamin D deficiency in smokers and nonsmokers), and use of vitamin D supplements.

### 2.4 Quality assessment

The quality assessment of the included studies used the Newcastle–Ottawa scale (NOS) based on cohort studies and case–control studies. We evaluated the article on the basis of selection, comparability, and outcome/exposure, with a possible total quality evaluation of 10. The Australian JBI Centre for Evidence‐Based Health Care (2016) Quality Assessment Tool was used as the evaluation criteria for the cross‐sectional study. All articles are evaluated by a reviewer.

### 2.5 Outcome measures

Our primary outcome measure was adult circulating vitamin D levels, compared between smokers and nonsmokers, and performed a subgroup analysis based on meta‐regression results.

### 2.6 Statistical analyses

Continuous results were calculated using Cohen's method as mean difference (SMD) and 95% confidence interval (CI), and the combined effect values of dichotomous variables were RR and its 95% confidence interval, using an inverse‐variance model. *I* ^2^ statistics were used to test heterogeneity among studies. A value of less than 50% of *I* ^2^ is considered low heterogeneity. In statistics, when there is significant heterogeneity, the size of the mixed effect is calculated by using the random effect model. Otherwise, a fixed effect model is used. Suppose *p* value < .05 or 95% CI without 0 (the 95% CI for the RR value does not include 1) was considered statistically significant. Based on the results of meta‐regression, the main sources of heterogeneity were determined and a subgroup analysis was conducted. The Begg method was used to assess publication bias. Data analysis was performed using STATA 14.

## 3 RESULTS

### 3.1 Characteristics of the chosen articles

**FIGURE 1:**
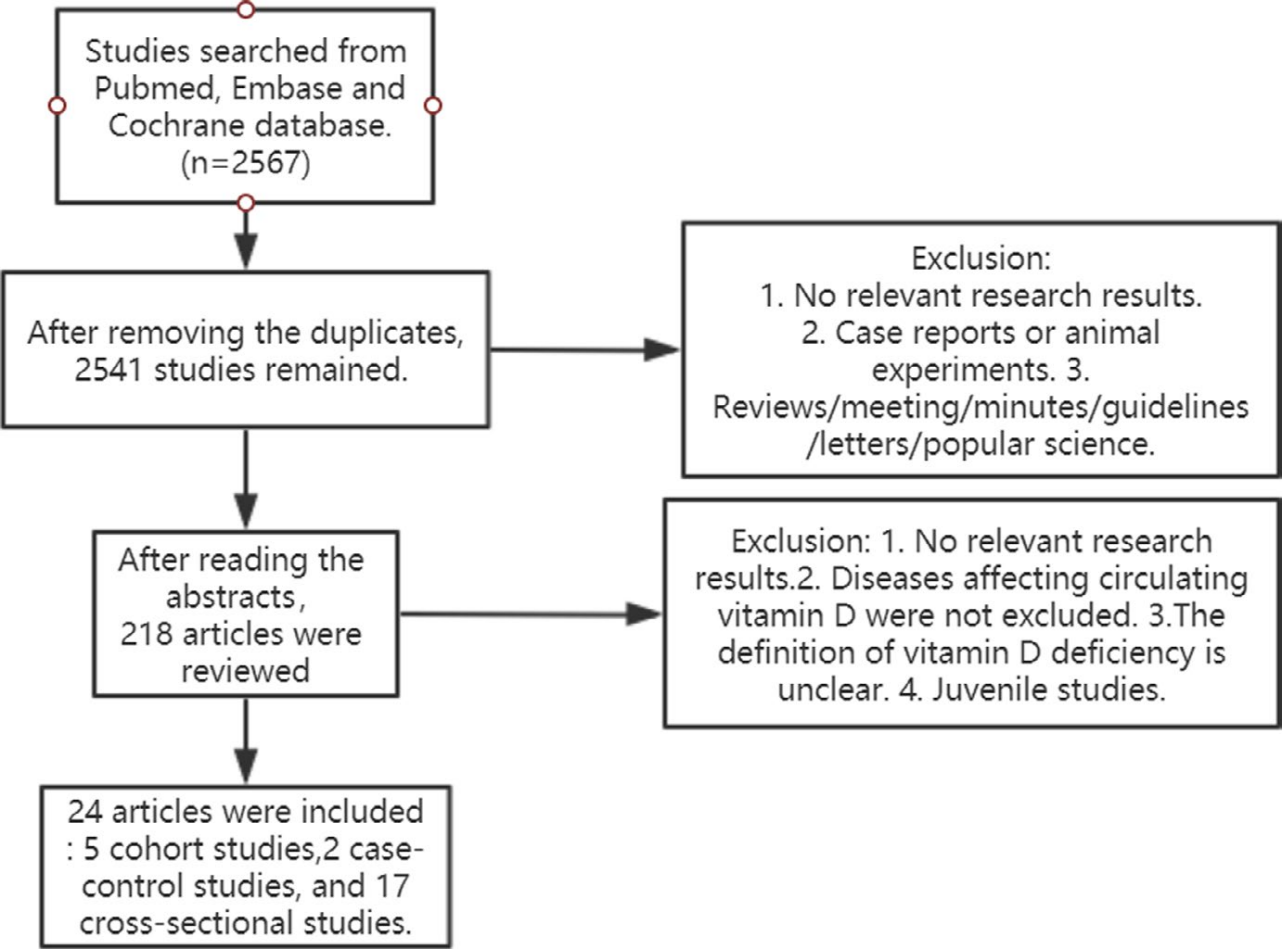
Flow chart

Two thousand five hundred forty‐one articles were retrieved from the database (Figure 1). After reviewing the summary, we selected 218 complete publications for further evaluation based on our inclusion criteria. Of these studies, 150 were excluded because patients with diseases that affect vitamin D absorption or metabolism were not excluded, and eight were excluded because the samples were not adult venous blood. There were 36 studies did not have the required data or did not contain sufficiently detailed data and that could not be obtained by contacting the authors. Finally, this meta‐analysis included 24 articles (Alam et al., 2018; Bianchi et al., 2012; Chaudhuri et al., 2013; Cuomo et al., 2019; Cutillas‐Marco et al., 2012; Díaz‐Gómez et al., 2007; Hermann et al., 2000; Jorde et al., 2019; Kassi et al., 2015; Kim et al., 2018; Klingberg et al., 2015; Li et al., 2016; Lokki et al., 2020; Lu et al., 2020; Mulligan et al., 2014; Okan et al., 2020; Richard et al., 2017; Schierbeck et al., 2012; Schmitt et al., 2018; Stürmer et al., 2015; Supervía et al., 2006; Tonnesen et al., 2016; Xu et al., 2020) (Table 1). The 24 studies involved 11,340 participants: 1,399 smokers and 9,941 nonsmokers were included in this meta‐analysis (when quitters are present, they are classified as nonsmokers and compared with current smokers and current nonsmokers). The following vitamin D detection methods were used in 24 selected articles: electrochemiluminescence immunoassay, chemiluminescent immunoassay, LC‐MS/MS, ELISA, RIA, and CMIA. Among them, two samples were plasma and the remaining 22 samples were serum. The specific characteristics of each study were shown in Table 1.

**TABLE 1:** Characteristics of included studies

Characteristics of dichotomous variable studies													
Author	Year	Type of experiment	Number of participants	Age (Mean)	BMI (Mean)	Detection method	Sample	Unit	Smokers with VD deficiency	Nonsmokers with VD deficiency	Smokers without VD deficiency	Nonsmokers without VD deficiency	Use of VD supplements
Sara Bianchi	2012	Cross‐sectional Study	185	60	25	CLIA	Serum	ng/ml	26	136	3	20	No
Aline Richard	2017	Cohort study	204	31.1	22.74	Elecsys	Serum	ng/ml	12	89	8	92	Yes
Rune Tønnesen	2016	Cross‐sectional Study	700	21.8	23.22	CLIA	Serum	nmol/L	139	234	94	233	No
F. Okan	2018	Cross‐sectional Study	72	73.5	27.8	Elecsys	Serum	ng/ml	11	48	1	12	No
Kuibao Li	2016	Cross‐sectional Study	348	62.4	26	ELISA	Serum	ng/ml	47	114	72	115	Yes
Eneida Boteon Schmitt	2017	Cross‐sectional Study	463	57.8	29.04	CLIA	Serum	ng/ml	47	268	18	130	No
Louise Lind Schierbeck	2012	Cohort study	2,013	50	25.11	RIA	Serum	nmol/L	369	419	462	763	Yes
Jaydip Ray Chaudhuri	2013	Cross‐sectional Study	150	49.4		CMIA	Serum	ng/ml	15	44	20	71	No
Sun Hea Kim	2018	Cross‐sectional Study	2,687	72.26	23.83	Unclear	Serum	ng/ml	237	1,432	135	883	Unclear
YaWen Lu	2020	Cross‐sectional Study	1,798	62	24.54	CLIA	Serum	ng/ml	76	528	255	939	Unclear
HaoWei Xu	2020	Cross‐sectional Study	232	65.5	23.34	Unclear	Serum	ng/ml	12	191	3	26	Yes
*Alessandro Cuomo*	2019	Cross‐sectional Study	290	47.8	26.2	Unclear	Serum	ng/ml	147	125	0	18	Unclear
*Giovanni Targher*	2006	Cross‐sectional Study	390	57.7	28.5	CLIA	Serum	nmol/L	30	100	44	216	No

Characteristics of continuous variable studies													
Author	Year	Type of experiment	Number of participants	Age (Mean)	BMI (Mean)	Detection method	Sample	Unit	Number of smoker	Number of nonsmoker	Smokers' VD (Mean ± *SD*)	Nonsmokers' VD (Mean ± *SD*)	Use of VD supplements
Michael Stürmer	2015	Cross‐sectional Study	146	57	27	Elecsys	Plasma	ng/ml	124	22	23.2 ± 9.4	22.3 ± 8.9	NO
A.P. Hermann	1999	Cohort study	2015	50.6		RIA	Serum	ng/ml	825	1,181	24.1 ± 12.1	25.8 ± 12.3	Unclear
N. Marta Díaz‐Gómez	2007	Cohort study	61	30.13	22.86	RIA	Serum	ng/ml	32	29	23.9 ± 8.7	29.9 ± 11.6	Yes
Eugenia Cutillas‐Marco	2012	Cross‐sectional Study	177	47	23.84	CLIA	serum	ng/ml	71	105	22.82 ± 10.33	24.66 ± 6.94	Yes
Eva N. Kassi	2014	Cross‐sectional Study	181	34.69	25.94	LC‐MS/MS	Serum	ng/ml	64	117	17 ± 6.83	21.36 ± 6.56	NO
A. Supervía	2005	Cross‐sectional Study	74	32.26	23.59	RIA	Serum	ng/ml	22	52	16.77 ± 9.9	31.94 ± 15.1	Unclear
Jennifer K. Mulligan	2014	Case–control study	21	46.29		ELISA	Plasma	ng/ml	9	12	25.32 ± 20.99	46.6 ± 16.18	NO
Muhammad Shah Alam	2018	Cross‐sectional Study	50	40.64	26.49	CLIA	Serum	ng/ml	21	29	27.1 ± 2.49	28.49 ± 6.38	NO
Klingberg, E	2015	Cross‐sectional Study	540	40.5	24.8	CLIA	Serum	nmol/L	25	515	51.4 ± 26.2	63.4 ± 26.2	Yes
A Inkeri Lokki	2018	Case–control study	359	27.06	24.12	CMIA	Serum	nmol/L	46	313	32.1 ± 14.6	41.6 ± 19.5	Unclear
Rolf Jorde	2019	Cohort study	406	51.9	27.8	Unclear	Serum	nmol/L	86	320	31 ± 12	34.8 ± 13	NO

### 3.2 Quality assessment

The quality of the studies was evaluated according to NOS and JBI standards. The literature in this meta‐analysis included five cohort studies, two case–control studies, and 17 cross‐sectional studies. We judged study quality in terms of selection, comparability, and results/exposure of studies according to the Newcastle–Ottawa scale of observational studies (Table 2). The total quality scores of the seven articles (cohort and case–control studies) ranged from 4 to 9, with a possible total of 10 points. The authenticity evaluation results of the cross‐sectional study according to the JBI quality evaluation tool are shown in Table 2. Due to strict inclusion and exclusion criteria to exclude the influence of certain diseases on circulating vitamin D levels, these studies had low selection bias. Since the purpose of this study was to explore the effects of smoking on circulating vitamin D levels in adults, the exposure factors were identified as smoking, and subject information was obtained using questionnaires or written self‐reports; no study using smoking as an exposure factor was subject blind.

**TABLE 2:** Quality evaluation of included studies

Cohort study and case–control study NOS (10)	Selectivity	Comparability	Outcome
A.P. Hermann	4	2	3
N. Marta Díaz‐Gómez	3	2	3
Jennifer K. Mulligan	2	1	1
A Inkeri Lokki	2	1	2
Aline Richard	3	2	2
Louise Lind Schierbeck	4	2	3
Rolf Jorde	3	2	2

Cross‐sectional study JBI (8)	Yes	No	Not clear
Michael Stürmer	5		3
Sara Bianchi	7		1
Eugenia Cutillas‐Marco	6		2
Eva N. Kassi	7		2
A. Supervía	4	2	2
Muhammad Shah Alam	7		1
Klingberg, E	6		2
Rune Tønnesen	6		2
F. Okan	4		4
Kuibao Li	7		1
Eneida Boteon Schmitt	7		1
Jaydip Ray Chaudhuri	7		1
Sun Hea Kim	6		2
Ya‐Wen Lu	6		2
HaoWei Xu	6		2
Alessandro Cuomo	5		3
Giovanni Targher	4	2	2

### 3.3 Vitamin D deficiency or insufficiency in smokers and nonsmokers in the dichotomous variable studies

**FIGURE 2:**
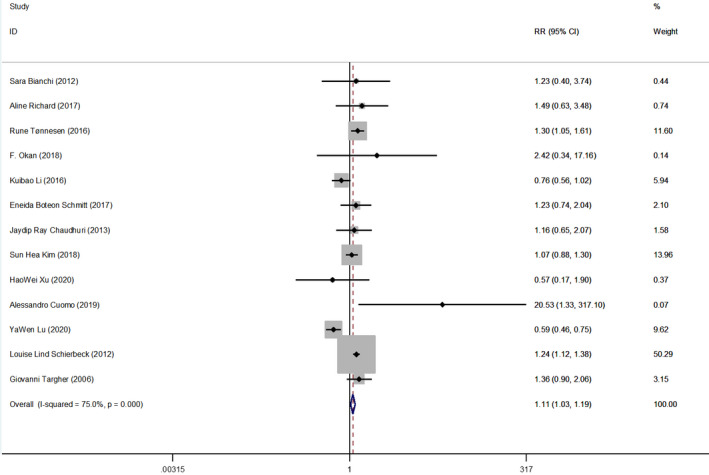
Vitamin D deficiency or insufficiency in adult smokers

**FIGURE 3:**
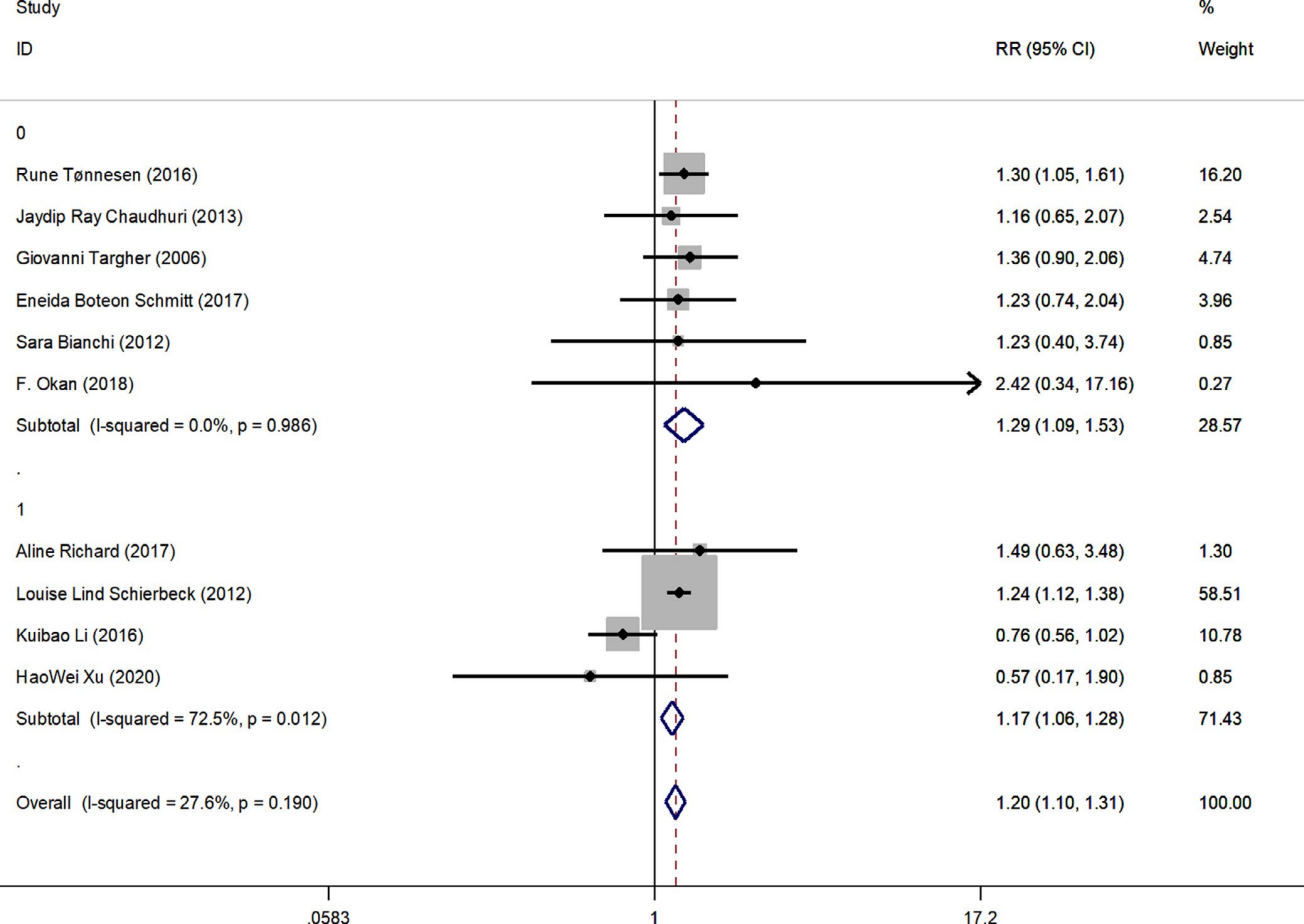
Vitamin D deficiency in adult smokers with or without vitamin D supplement

People who are current smokers are more likely to have circulating vitamin D deficiency or deficiency than nonsmokers. We obtained a 95% confidence interval combined effect amount (RR 1.11, 95% CI: 1.03–1.19, *p* < .001). *p* < .05 and 95% CI did not contain 1. The model is fixed, inverse‐variance, *I* ^2^ = 75% (Figure 2). No source of heterogeneity was found in meta‐regression. Sensitivity analysis suggested that study by Yawen Lu et al. was the main source of heterogeneity. When that study was removed, *I* ^2^ dropped to 38%. To better rule out the effect of vitamin D supplement use on the results, we analyzed the use of vitamin D supplements in subgroups. The use of vitamin D supplements was defined as 1, and nonuse of vitamin D supplements was defined as 0. The results are as follows (Figure 3): The combined effect size was available in people who did not receive vitamin D supplementation (RR 1.29, 95% CI: 1.09–1.53, *p* < .05), *I* ^2^ = 0 in this subgroup. A combined effect size was obtained when vitamin D supplements were known to be used (RR1.17, 95% CI: 1.06–1.28, *p* < .05), *I* ^2^ = 72.5% in this subgroup.

### 3.4 Circulating vitamin D levels in smokers and nonsmokers in continuous variable studies

**FIGURE 4:**
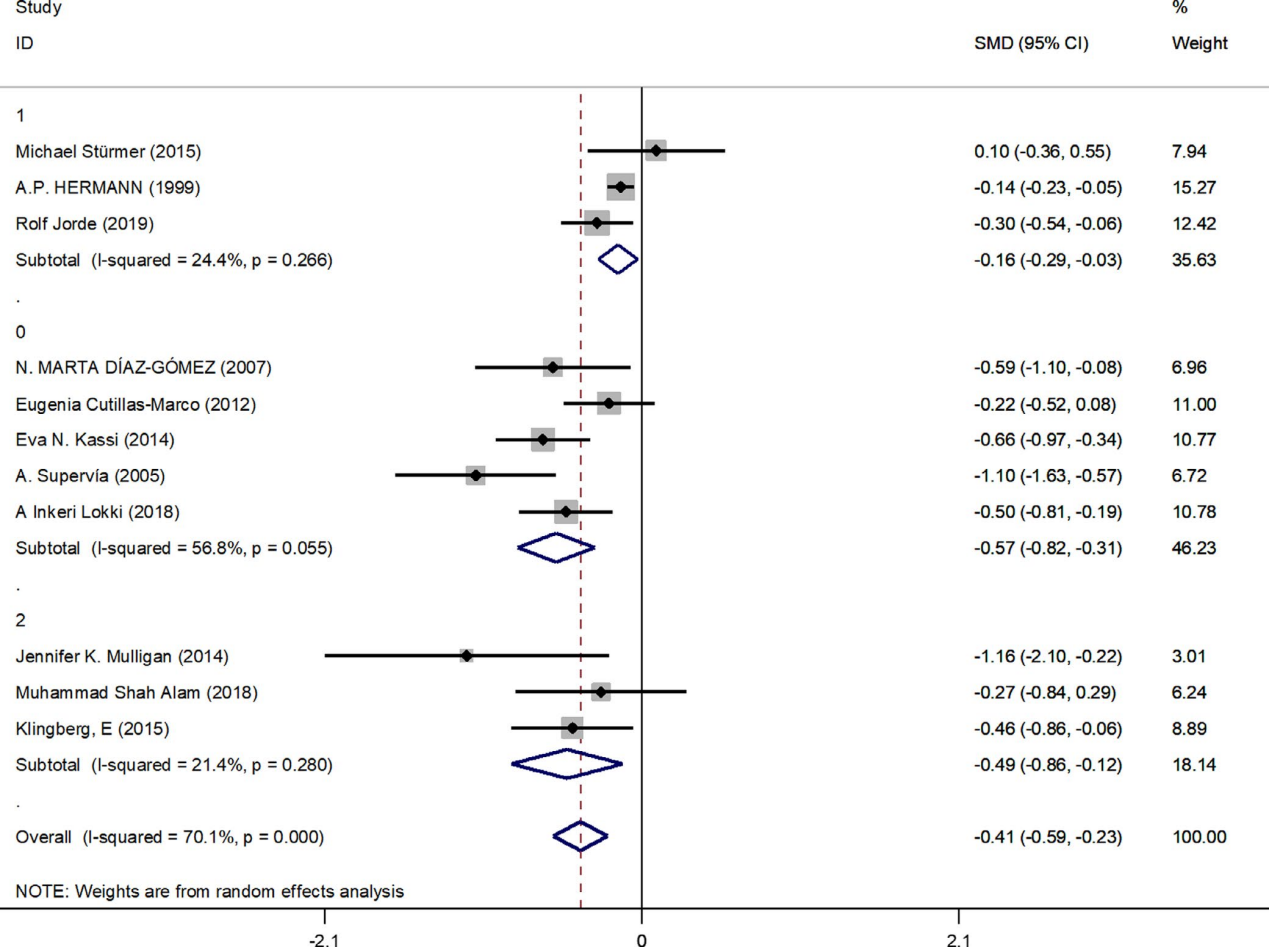
Vitamin D levels of smokers at different ages

People who are current smokers had lower circulating vitamin D levels than nonsmokers. Due to the different vitamin D units provided in the literature, the combined effect amount was expressed by SMD. We obtained a 95% CI combined effect amount (SMD −0.24, 95% CI: −0.31 to −0.17, *p* < .001). *p* < .05 and 95% CI did not contain 0, which was statistically significant. *I* ^2^ = 70.1%. Due to the large heterogeneity, REML method was used to conduct univariate meta‐regression analysis one by one with the number of subjects, type of experiment, year of publication, average age, test method, sample type, and use of vitamin D supplement as covariables, and the combined effect amount as dependent variables. The results are as follows (Table 3): Meta‐regression results suggested that age was the main source of heterogeneity (Coef. 0.022, 95% CI: 1.009–1.037, *p* < .05). Consider that most women reach menopause around the age of 50. Bone metabolism in premenopause and postmenopause is affected by fluctuation of estrogen level, and circulating vitamin D level may change accordingly. We divided the study into three subgroups with mean age over 50, 40–50 years old (excluding 50), and under 40 years old (excluding 40) as the dividing line, and the results are as follows (Figure 4). In the over 50 group (group 1), we get a combined effect size (SMD‐0.16, 95% CI: −0.29 to −0.03, *p* < .05), intragroup heterogeneity *I* ^2^ = 24.4%. In 40‐ to 50‐year‐old group (group 2), we get a combined effect size (SMD‐0.49, 95% CI: −0.86 to −0.12, *p* < .05), intragroup heterogeneity *I* ^2^ = 21.4%. Under 40‐year‐old group (group 0) to get a combined effect size (SMD‐0.57, 95% CI: −0.82 to −0.31, *p* < .05), intragroup heterogeneity *I* ^2^ = 56.8%.

**TABLE 3:** Meta‐regression results of continuous variables

	Subjects	Age	Methods	Supplement	Research type	Year	Sample
Coef.	<0.001	0.022	−0.031	−0.467	−0.055	<0.001	0.176
*p*	.209	.005	.475	.714	.714	.983	.603

### 3.5 Publication bias

Begg's test was used to examine the publication bias of the literature. Adjustment statistics of dichotomous variable studies *Z* = 0.18, *p* = .855, *p* > .05. Adjustment statistics of continuous variable studies *Z* = 1.4, *p* = .161, *p* > .05. There was no publication bias in the studies of dichotomous variables and continuous variables.

## 4 DISCUSSION

This meta‐analysis provides evidence of the negative effects of smoking on circulating vitamin D levels. These results summarized the findings from some small samples, the meta‐analysis showed that smoking was associated with lower levels of circulating vitamin D, and the influence between smokers and nonsmokers and whether to use vitamin D supplements no correlation, because no matter whether to use vitamin D supplements, the results of the meta‐analysis indicate current smokers were more likely than nonsmokers to circulating vitamin D deficiency or inadequate. In the group without vitamin D supplement, *I* ^2^ = 0, intragroup heterogeneity did not exist; while in the group with vitamin D supplement, *I* ^2^ = 72.5%, intragroup heterogeneity was high. It may be related to the differences in the dose and dosage form of vitamin D taken by study participants, or it may be related to the small number of studies included in the group. In the study of continuous variables, we preliminarily obtained a result with high heterogeneity (*I* ^2^ = 70.1%). After meta‐regression, it was known that age was the main factor causing heterogeneity. We divided different age groups into different subgroups for analysis. We obtained two results with low heterogeneity in the over‐50 group and the 40–50 group and one result with moderate heterogeneity in the under‐40 group. Since most women of childbearing age are under the age of 40, we hypothesized that the source of heterogeneity might be the use of contraceptives by women of childbearing age, resulting in an impact on the results. Circulating vitamin D levels in smokers were lower than in nonsmokers at different ages, and this was particularly evident in the under 40 age group and on the surface that the effect of smoking on circulating vitamin D levels decreases with age. But studies have shown that smoking has the greatest effect on bones in older people, while no significant effect has been observed in any part of the bones in people under 40 (Ward & Klesges, 2001). The effect of smoking on bone metabolism is greater in the elderly than in the young, and the dose effect of smoking on bone has been shown in some literature (McCulloch et al., 1991; Ortego‐Centeno et al., 1997; Välimäki et al., 1994). The same phenomenon has been seen in studies of smoking and circulating vitamin D levels (Rapuri et al., 2000). This is contrary to our observation that smoking has a greater negative effect on circulating vitamin D levels in young people. This may have something to do with the fact that older people take vitamin D supplements at a higher rate than younger people, although there is no clear evidence to support this conclusion. However, after the exclusion of vitamin D supplements, the RR for smoking‐induced vitamin D deficiency increased roughly with age.

Many studies have explored the impact of smoking on vitamin D endocrine system (VDES). Previous studies found that smoking can affect vitamin D metabolism in many ways, including vitamin D intake, synthesis, hydroxylation, and catabolism. Tobacco smoke contains substances such as polycyclic aromatic hydrocarbons, aldehydes, and DDT and is a carcinogen, neurotoxin, and endocrine disruptor (Diamanti‐Kandarakis et al., 2009; Smith & Hansch, 2000). There are several hypotheses about the specific mechanism by which smoking lowers vitamin D levels.

Skin synthesis is the main source of vitamin D in the human body, and vitamin D synthesis is affected by skin aging. Smoking (Ernster et al., 1995) and ultraviolet radiation from the sun (Pillai et al., 2005) are considered important factors that contribute to skin aging in humans. Photosynthesis of precholecalciferol in human skin, that is, solar ultraviolet B photons with energy between 290 and 315 nanometer penetrates the skin under sunlight, making 7‐dehydrogenated cholesterol (provitamin D3)(7‐DHC) photolysis into precholecalciferol (provitamin D) (

**FIGURE 5:**
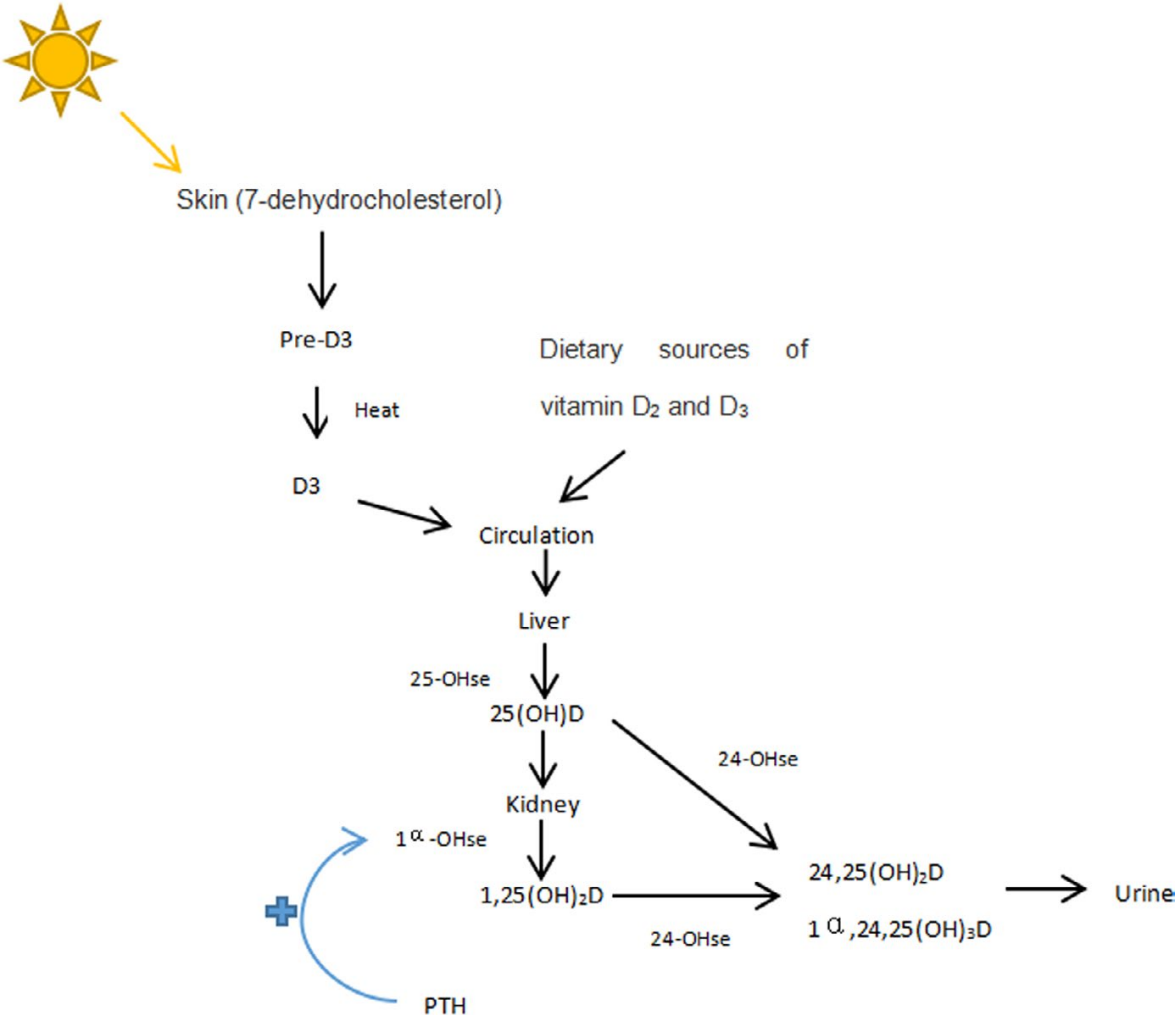
Endocrine system of vitamin D

Figure 5). The precholecalciferol is thermodynamically unstable and requires isomerization to form the cholecalciferol. Once formed, cholecalciferol passes from the skin into the bloodstream, where it binds to vitamin D‐binding proteins (Holick et al., 1980; Kira et al., 2003; MacLaughlin et al., 1982). Increased skin aging significantly reduces the skin's ability to convert 7‐DHC to precholecalciferol (Holick, 1995). López Hernández et al. (1995) found evidence of accelerated skin aging among smokers, finding that smoking, sun exposure, and age are important factors that increase skin wrinkling, and skin wrinkles are a significant sign of skin aging. Matrix metalloproteinases (MMPs) induce photoaging, and there is ample evidence to emphasize the effect of smoking on skin aging by activating MMPs (Holick, 1995; Lahmann et al., 2001). This is consistent with our findings that in people who do not take vitamin D supplements, the risk of vitamin D deficiency in smokers increases with age. Smoking can also affect the expression of cytokines and inflammatory mediators. In the study of Tsutakawa et al. (2009), it was found that nicotine (0.35 mg kg^‐1^ day^‐1^) significantly enhanced the protein expression of cyclooxygenase 2(COX‐2) and inducible nitric oxide synthase (iNOS), increased the release of pro‐inflammatory mediators, and caused damage or delayed healing of skin and blood vessels.

On the other hand, the decrease in vitamin D may be related to the inhibition of PTH caused by smoking. Several studies we included (Cutillas‐Marco et al., 2012; Díaz‐Gómez et al., 2007; Supervía et al., 2006) detected parathyroid hormone levels in smokers and found that PTH levels were lower in smokers than in nonsmokers. Parathyroid hormone levels are mainly regulated by calcium ions. In the study by Need et al. (2002) (Schwarz et al., 1994), smokers had higher levels of ionized calcium in their serum, and small changes in ionized calcium levels caused rapid changes in parathyroid hormone secretion and synthesis. Several other hypothesized regulators, such as chromium‐granin peptides and interleukin‐8, may also be involved in regulating PTH secretion (Jorde et al., 2005). In addition, smoke may have a direct toxic effect on parathyroid cells. Jorde et al. (2005) hypothesized that there may be some substances in cigarette smoke that interact directly with calcium receptors, allowing smoking to enhance the degradation of parathyroid hormone in blood samples, leading to a decrease in parathyroid hormone levels without affecting calcium homeostasis. Studies have shown that low serum concentrations of VD and the dose–response pattern of smoking, that is, smoking for longer periods of time and smoking more, lead to lower levels of vitamin D (Hermann et al., 2000; Jiang et al., 2016). Although studies have suggested that current smokers have lower levels of parathyroid hormone than nonsmokers (Brot et al., 1999; Jorde et al., 2019; Landin‐Wilhelmsen et al., 1995; Paik et al., 2010), it is not clear whether this dose relationship exists. But both parathyroid hormone and vitamin D levels are similar to those of never smokers after smoking cessation (Jorde et al., 2005). We conjectured that decreased vitamin D levels in smokers may be associated with decreased parathyroid hormone secretion, since PTH enhances the effect of renal 1‐hydroxylase on vitamin D3 activation (Lips, 2001).

In addition, vitamin D3 synthesized in the skin and vitamin D2 extracted from food circulating in the body are first hydroxylated as they pass through the liver and then hydroxylated by the 1α hydroxylase CYP27B1 as they pass through the kidneys to form active vitamin D,1,25(OH)2D (Omdahl et al., 2002). Long‐term smoking can lead to accumulation of heavy metals in the body. Tobacco plants contain lead and cadmium (Lugon‐Moulin et al., 2006). The decrease in serum 1,25(OH)2D levels in smokers is related to the accumulation of cadmium in the kidneys (Brot et al., 1999; Kido et al., 1989). The mechanism of this process may be renal damage caused by cadmium and lead poisoning. In fact, the increase in lead and cadmium in the body can damage renal tubular function and glomerular function (Cooper, 2006).

Smoking can also lead to disorders in the catabolism of vitamin D. A polycyclic aromatic hydrocarbon, benzopyrene (BaP) in tobacco smoke increases the recruitment of CYP24A1 promoters by 1,25(OH) 2D3‐dependent VDR and retinoid X receptors, which promotes the decomposition of 1,25(OH)2D3 in human monocyte/macrophage‐derived THP‐1 cells and reduces vitamin D levels by enhancing induced expression of cytochrome P450 24A1(CYP24A1) (Matsunawa et al., 2009). Another arene receptor (AhR) ligand, 2,3,7,8‐tetrachlorodibenzo‐dioxins, enhances CYP24A1 expression of 1,25(OH)2D3 in THP‐1 cells. After treatment with AhR antagonists and protein synthesis inhibitors, the enhancement of CYP24A1 induced by BaP was inhibited, indicating that the role of BaP was mediated by AhR activation and de novo synthesis of proteins. Therefore, the activation of AhR by BaP can promote catabolism of vitamin D3 (Matsunawa et al., 2009). Other researchers have found that SNP rs4809957 located in the three untranslated regions of CYP24A1 gene 20q13.2 interacts with smoking, and 1,25(OH)2D3 plays an antiproliferation role on vitamin D receptor‐mediated human cancer cells (Dong et al., 2012). When smokers' CYP24A1 genes were affected by smoke, lower vitamin D levels were linked to lung cancer risk.

Other studies have shown that tobacco alters the sense of smell and taste of food, leading to lower levels of vitamin D intake (Frye et al., 1990; Grunberg, 1982; Morabia et al., 2000; Redington, 1984). It has been reported that other peripheral tissues, including respiratory epithelial cells, also contain 1a‐hydroxylase and may serve as a source of 1,25VD3 (Hansdottir et al., 2008). The researchers found that acrolein and extract (CSE) from tobacco smoke significantly reduced the conversion of 25(OH)D3 to 1,25(OH)2D3 in airway epithelial cells by downregulating the expression of CYP27B1(CYP27B1 is the gene encoding the 25‐hydroxyvitamin D3 1a hydroxylase responsible for converting 25VD3 to 1,25(OH)2D3) (Mulligan et al., 2014). One of the articles we included was a study of patients with chronic rhinosinusitis (Mulligan et al., 2014), showed that smokers, regardless of whether they had chronic rhinosinusitis, had lower circulating vitamin D levels than nonsmokers.

This study is the first meta‐analysis of circulating vitamin D levels in smokers. There have been many independent studies, but this is the first time we have combined statistics on this subject. Because diseases affecting vitamin D absorption or metabolism were excluded in advance, the results of this meta‐analysis were not affected by adverse or beneficial effects of disease on vitamin D sources and metabolism. Confounding factors such as estrogen replacement therapy may partially mask the effect on women, and oral contraceptives or estrogen may prevent bone loss, increasing error variability. So we also excluded menopausal women who were on hormone replacement therapy. However, for women of childbearing age using oral contraceptives, it is unclear whether these women of childbearing age using contraceptives have been excluded since no such information was provided in the article, which may be one of the sources of heterogeneity in the subgroup of this population. In addition, several limitations of our study must be noted. Since vitamin D samples were taken from serum and plasma, this may lead to interstudy heterogeneity. However, meta‐regression showed that the sample type was not the main source of interstudy heterogeneity, which had little impact on the results, and the few studies with the sample type of plasma were not suitable for further subgroup analysis. More research is needed to confirm whether vitamin D levels in smokers are reduced in serum and plasma, respectively. Our study did not analyze the effect of exercise on VD levels. Some studies have suggested that physical activity has an effect on vitamin D levels, but studies until now have not established a direct relationship between physical activity and VD (Bell et al., 1988; Jacques et al., 1997; Klausen et al., 1993; Lucas et al., 2005; Ma Moun et al., 2005; Scragg et al., 1992, 1995), or that sun exposure is a confounding factor, and there are conflicting results regarding the effect of physical activity on 25(OH)D metabolism. It was found that there was no significant relationship between physical activity and 25(OH)D after controlling for sunshine duration. In addition, since the included studies came from different countries and regions, and the included studies were mainly retrospective studies, the time, season, and latitude of blood sample extraction of subjects could not be unified, and these confounding factors might affect the results and produce heterogeneity among studies. However, in a single study, the subjects had the same season, latitude, and blood sampling time, and there were relevant measures to exclude the influence of confounding factors, so the study results were less affected by these factors. The association between smoking and circulating vitamin D levels is unlikely to be accidental, as estimates come from a large mixed sample (more than 11,340 participants) and these studies are heterogeneous in terms of several participant and methodological characteristics. Due to the exclusion of some diseases that affect vitamin D absorption or metabolism, there is inevitably a selection bias, and since all studies rely on self‐reporting, there may be bias in determining smoking exposure.

Smoking‐related vitamin D deficiency (VDD) may pose an even greater public health problem in our aging society. Given the cumulative and dose‐dependent effects of smoking on VDD and its association with the vitamin D‐PTH axis, an increase in smoking among these young populations is likely to lead to a significant increase in the future public health burden of osteoporosis or other cardiovascular, pulmonary, infectious, and immune‐related diseases. Smoking has a significant adverse effect on circulating vitamin D levels, but vitamin D levels can be restored to nonsmoker levels after quitting smoking. In view of the adverse effects of smoking on vitamin D‐PTH axis health and its associated public effects on bone metabolism and other systemic health, the prevention of VDD and smoking cessation must be emphasized.

## CONFLICT OF INTEREST

The authors declare that there is no conflict of interest.

## AUTHOR CONTRIBUTIONS

**Lu Yang:** Formal analysis (lead); Writing‐original draft (equal). **Hang Zhao:** Formal analysis (supporting); Methodology (equal). **Ke Liu:** Investigation (equal). **Yichao Wang:** Investigation (equal). **Tiantian Sun:** Investigation (equal). **Qianqian Liu:** Investigation (equal). **Shuchun Chen:** Writing‐review & editing (equal). **Luping Ren:** Writing‐review & editing (equal).

## Data Availability

The data that supports the findings of this study are available in this article. No additional supplementary material is provided.

## References

[fsn32488-bib-0001] Alam, M. S. , Kamrul‐Hasan, M. , Kalam, S. T. , Selim, S. , Akter, F. , & Saifuddin, M. (2018). Vitamin D status in newly diagnosed type 2 diabetes patients attending in a tertiary hospital of Bangladesh. Mymensingh Medical Journal, 27, 362–368. 29769503

[fsn32488-bib-0002] Bell, N. H. , Godsen, R. N. , Henry, D. P. , Shary, J. , & Epstein, S. (1988). The effects of muscle‐building exercise on vitamin D and mineral metabolism. Journal of Bone and Mineral Research, 3, 369–373. 10.1002/jbmr.5650030402 3265576

[fsn32488-bib-0003] Berridge, M. J. (2017). Vitamin D deficiency and diabetes. Biochemical Journal, 474, 1321–1332. 10.1042/BCJ20170042 28341729

[fsn32488-bib-0004] Bianchi, S. , Maffei, S. , Prontera, C. , Battaglia, D. , & Vassalle, C. (2012). Preanalytical, analytical (DiaSorin LIAISON) and clinical variables potentially affecting the 25‐OH vitamin D estimation. Clinical Biochemistry, 45, 1652–1657. 10.1016/j.clinbiochem.2012.08.003 22906830

[fsn32488-bib-0005] Boulet, L.‐P. , Catherine, L. , Francine, A. , Guy, C. , Descary, M. C. , & Francine, D. (2006). Smoking and asthma: Clinical and radiologic features, lung function, and airway inflammation. Chest, 129, 661–668. 10.1378/chest.129.3.661 16537865

[fsn32488-bib-0006] Brot, C. , Jorgensen, N. R. , & Sorensen, O. H. (1999). The influence of smoking on vitamin D status and calcium metabolism. European Journal of Clinical Nutrition, 53, 920–926. 10.1038/sj.ejcn.1600870 10602348

[fsn32488-bib-0007] Chaudhuri, J. R. , Mridula, K. R. , Anamika, A. , Boddu, D. B. , Misra, P. K. , Lingaiah, A. , Balaraju, B. , & Bandaru, V. S. (2013). Deficiency of 25‐hydroxyvitamin d and dyslipidemia in Indian subjects. Journal of Lipids, 2013, 623420. 10.1155/2013/623420 24455278 PMC3880703

[fsn32488-bib-0008] Cooper, R. G. (2006). Effect of tobacco smoking on renal function. Indian Journal of Medical Research, 124, 261–268. 17085829

[fsn32488-bib-0009] Cuomo, A. , Maina, G. , Bolognesi, S. , Rosso, G. , Beccarini Crescenzi, B. , Zanobini, F. , Goracci, A. , Facchi, E. , Favaretto, E. , Baldini, I. , Santucci, A. , & Fagiolini, A. (2019). Prevalence and correlates of vitamin D deficiency in a sample of 290 inpatients with mental illness. Frontiers in Psychiatry, 10, 167. 10.3389/fpsyt.2019.00167 31001150 PMC6455075

[fsn32488-bib-0010] Cutillas‐Marco, E. , Fuertes‐Prosper, A. , Grant, W. B. , & Morales‐Suárez‐Varela, M. (2012). Vitamin D deficiency in South Europe: Effect of smoking and aging. Photodermatology, Photoimmunology & Photomedicine, 28, 159–161. 10.1111/j.1600-0781.2012.00649.x 22548399

[fsn32488-bib-0011] Diamanti‐Kandarakis, E. , Bourguignon, J. P. , Giudice, L. C. , Hauser, R. , Prins, G. S. , Soto, A. M. , Zoeller, R. T. , & Gore, A. C. (2009). Endocrine‐disrupting chemicals: An Endocrine Society scientific statement. Endocrine Reviews, 30, 293–342. 10.1210/er.2009-0002 19502515 PMC2726844

[fsn32488-bib-0012] Díaz‐Gómez, N. M. , Mendoza, C. , González‐González, N. L. , Barroso, F. , Jiménez‐Sosa, A. , Domenech, E. , Clemente, I. , Barrios, Y. , & Moya, M. (2007). Maternal smoking and the vitamin D‐parathyroid hormone system during the perinatal period. The Journal of Pediatrics, 151, 618–623. 10.1016/j.jpeds.2007.05.003 18035141

[fsn32488-bib-0013] Dong, J. , Hu, Z. , Wu, C. , Guo, H. , Zhou, B. , Lv, J. , Lu, D. , Chen, K. , Shi, Y. , Chu, M. , Wang, C. , Zhang, R. , Dai, J. , Jiang, Y. , Cao, S. , Qin, Z. , Yu, D. , Ma, H. , Jin, G. , … Shen, H. (2012). Association analyses identify multiple new lung cancer susceptibility loci and their interactions with smoking in the Chinese population. Nature Genetics, 44, 895–899. 10.1038/ng.2351 22797725 PMC6628171

[fsn32488-bib-0014] Ernster, V. L. , Grady, D. , Miike, R. , Black, D. , Selby, J. , & Kerlikowske, K. (1995). Facial wrinkling in men and women, by smoking status. American Journal of Public Health, 85, 78–82. 10.2105/AJPH.85.1.78 7832266 PMC1615259

[fsn32488-bib-0015] Frye, R. E. , Schwartz, B. S. , & Doty, R. L. (1990). Dose‐related effects of cigarette smoking on olfactory function. JAMA, 263, 1233–1236. 10.1001/jama.1990.03440090067028 2304239

[fsn32488-bib-0016] Girgis, C. M. , Clifton‐Bligh, R. J. , Hamrick, M. W. , Holick, M. F. , & Gunton, J. E. (2013). The roles of vitamin D in skeletal muscle: Form, function, and metabolism. Endocrine Reviews, 34, 33–83. 10.1210/er.2012-1012 23169676

[fsn32488-bib-0017] Grunberg, N. E. (1982). The effects of nicotine and cigarette smoking on food consumption and taste preferences. Addictive Behaviors, 7, 317–331. 10.1016/0306-4603(82)90001-6 7183186

[fsn32488-bib-0018] Hansdottir, S. , Monick, M. M. , Hinde, S. L. , Lovan, N. , Look, D. C. , & Hunninghake, G. W. (2008). Respiratory epithelial cells convert inactive vitamin D to its active form: Potential effects on host defense. The Journal of Immunology, 181, 7090–7099. 10.4049/jimmunol.181.10.7090 18981129 PMC2596683

[fsn32488-bib-0019] Hermann, A. P. , Brot, C. , Gram, J. , Kolthoff, N. , & Mosekilde, L. (2000). Premenopausal smoking and bone density in 2015 perimenopausal women. Journal of Bone and Mineral Research, 15, 780–787. 10.1359/jbmr.2000.15.4.780 10780870

[fsn32488-bib-0020] Holick, M. F. (1995). Environmental factors that influence the cutaneous production of vitamin D. The American Journal of Clinical Nutrition, 61, 638S–645S. 10.1093/ajcn/61.3.638S 7879731

[fsn32488-bib-0021] Holick, M. F. , MacLaughlin, J. A. , Clark, M. B. , Holick, S. , Potts, J. , Anderson, R. , Blank, I. , Parrish, J. , & Elias, P. (1980). Photosynthesis of previtamin D3 in human skin and the physiologic consequences. Science, 210, 203–205. 10.1126/science.6251551 6251551

[fsn32488-bib-0022] Jääskeläinen, T. , Knekt, P. , Marniemi, J. , Sares‐Jäske, L. , Männistö, S. , Heliövaara, M. , & Järvinen, R. (2013). Vitamin D status is associated with sociodemographic factors, lifestyle and metabolic health. European Journal of Nutrition, 52, 513–525. 10.1007/s00394-012-0354-0 22538929

[fsn32488-bib-0023] Jacques, P. F. , Felson, D. T. , Tucker, K. L. , Mahnken, B. , Wilson, P. W. , Rosenberg, I. H. , & Rush, D. (1997). Plasma 25‐hydroxyvitamin D and its determinants in an elderly population sample. The American Journal of Clinical Nutrition, 66, 929–936. 10.1093/ajcn/66.4.929 9322570

[fsn32488-bib-0024] Jiang, C. Q. , Chan, Y. H. , Xu, L. , Jin, Y. L. , Zhu, T. , Zhang, W. S. , Cheng, K. K. , & Lam, T. H. (2016). Smoking and serum vitamin D in older Chinese people: Cross‐sectional analysis based on the Guangzhou Biobank Cohort Study. British Medical Journal Open, 6, e10946. 10.1136/bmjopen-2015-010946 PMC4932269 27338881

[fsn32488-bib-0025] Jorde, R. , Saleh, F. , Figenschau, Y. , Kamycheva, E. , Haug, E. , & Sundsfjord, J. (2005). Serum parathyroid hormone (PTH) levels in smokers and non‐smokers. The fifth Troms ø study. European Journal of Endocrinology, 152, 39–45. 10.1530/eje.1.01816 15762185

[fsn32488-bib-0026] Jorde, R. , Stunes, A. K. , Kubiak, J. , Grimnes, G. , Thorsby, P. M. , & Syversen, U. (2019). Smoking and other determinants of bone turnover. PLoS One, 14, e225539. 10.1371/journal.pone.0225539 PMC6876776 31765401

[fsn32488-bib-0027] Kanis, J. A. , Johnell, O. , Oden, A. , Johansson, H. , De Laet, C. , Eisman, J. A. , Fujiwara, S. , Kroger, H. , McCloskey, E. V. , Mellstrom, D. , Melton, L. J. , Pols, H. , Reeve, J. , Silman, A. , & Tenenhouse, A. (2005). Smoking and fracture risk: A meta‐analysis. Osteoporosis International, 16, 155–162. 10.1007/s00198-004-1640-3 15175845

[fsn32488-bib-0028] Kassi, E. N. , Stavropoulos, S. , Kokkoris, P. , Galanos, A. , Moutsatsou, P. , Dimas, C. , Papatheodorou, A. , Zafeiris, C. , & Lyritis, G. (2015). Smoking is a significant determinant of low serum vitamin D in young and middle‐aged healthy males. Hormones, 14, 245–250. 25402376 10.14310/horm.2002.1521

[fsn32488-bib-0029] Kido, T. , Nogawa, K. , Yamada, Y. , Honda, R. , Tsuritani, I. , Ishizaki, M. , & Yamaya, H. (1989). Osteopenia in inhabitants with renal dysfunction induced by exposure to environmental cadmium. International Archives of Occupational and Environmental Health, 61, 271–276. 10.1007/BF00381425 2722250

[fsn32488-bib-0030] Kim, S. H. , Oh, J. E. , Song, D. W. , Cho, C. Y. , Hong, S. H. , Cho, Y. J. , Yoo, B. W. , Shin, K. S. , Joe, H. , Shin, H. S. , & Son, D. Y. (2018). The factors associated with Vitamin D deficiency in community dwelling elderly in Korea. Nutrition Research and Practice, 12, 387–395. 10.4162/nrp.2018.12.5.387 30323906 PMC6172170

[fsn32488-bib-0031] Kira, M. , Kobayashi, T. , & Yoshikawa, K. (2003). Vitamin D and the skin. The Journal of Dermatology, 30, 429–437. 10.1111/j.1346-8138.2003.tb00412.x 12810989

[fsn32488-bib-0032] Klausen, T. , Breum, L. , Sørensen, H. A. , Schifter, S. , & Sonne, B. (1993). Plasma levels of parathyroid hormone, vitamin D, calcitonin, and calcium in association with endurance exercise. Calcified Tissue International, 52, 205–208. 10.1007/BF00298719 8481833

[fsn32488-bib-0033] Klingberg, E. , Oler, D. G. , Konar, J. , Petzold, M. , & Hammarsten, O. (2015). Seasonal variations in serum 25‐hydroxy vitamin D levels in a Swedish cohort. Endocrine, 49, 800–808. 10.1007/s12020-015-0548-3 25681052 PMC4512566

[fsn32488-bib-0034] Lahmann, C. , Bergemann, J. , Harrison, G. , & Young, A. R. (2001). Matrix metalloproteinase‐1 and skin ageing in smokers. The Lancet, 357, 935–936. 10.1016/S0140-6736(00)04220-3 11289356

[fsn32488-bib-0035] Landin‐Wilhelmsen, K. , Wilhelmsen, L. , Lappas, G. , Rosén, T. , Lindstedt, G. , Lundberg, P. A. , Wilske, J. , & Bengtsson, B.‐A. (1995). Serum intact parathyroid hormone in a random population sample of men and women: Relationship to anthropometry, life‐style factors, blood pressure, and vitamin D. Calcified Tissue International, 56, 104–108. 10.1007/BF00296339 7736316

[fsn32488-bib-0036] Li, K. , Yang, X. , Wang, L. , Chen, M. , Zhao, W. , Xu, L. I. , & Yang, X. (2016). Modification of the association between smoking status and severity of coronary stenosis by vitamin D in patients suspected of coronary heart disease. Medicine, 95, e4817. 10.1097/MD.0000000000004817 27603397 PMC5023920

[fsn32488-bib-0037] Lips, P. (2001). Vitamin D deficiency and secondary hyperparathyroidism in the elderly: Consequences for bone loss and fractures and therapeutic implications. Endocrine Reviews, 22, 477–501. 10.1210/edrv.22.4.0437 11493580

[fsn32488-bib-0038] Liu, P. T. , Stenger, S. , Tang, D. H. , Modlin, R. L. (2007). Cutting edge: Vitamin D‐mediated human antimicrobial activity against Mycobacterium tuberculosis is dependent on the induction of cathelicidin. The Journal of Immunology, 179, 2060–2063. 17675463 10.4049/jimmunol.179.4.2060

[fsn32488-bib-0039] Lokki, A. I. , Heikkinen‐Eloranta, J. , Öhman, H. , Heinonen, S. , Surcel, H.‐M. , & Nielsen, H. S. (2020). Smoking during pregnancy reduces vitamin D levels in a Finnish birth register cohort. Public Health Nutrition, 23, 1273–1277. 10.1017/S1368980018003932 30732669 PMC10200425

[fsn32488-bib-0040] López Hernández, B. , Tercedor, J. , Ródenas, J. M. , Simón López, F. , Ortega del Olmo, R. M. , & Serrano Ortega, S. (1995). Skin aging and smoking. Revista Clínica Española, 195, 147–149. 7754147

[fsn32488-bib-0041] Lu, Y. W. , Chou, R. H. , Liu, L. K. , Chen, L.‐K. , Huang, P.‐H. , & Lin, S.‐J. (2020). The relationship between circulating vitamin D3 and subclinical atherosclerosis in an elderly Asian population. Scientific Reports, 10, 18704. 10.1038/s41598-020-75391-0 33127933 PMC7603322

[fsn32488-bib-0042] Lucas, J. A. , Bolland, M. J. , Grey, A. B. , Ames, R. W. , Mason, B. H. , Horne, A. M. , Gamble, G. D. , & Reid, I. R. (2005). Determinants of vitamin D status in older women living in a subtropical climate. Osteoporosis International, 16, 1641–1648. 10.1007/s00198-005-1888-2 16027959

[fsn32488-bib-0043] Lucato, P. , Solmi, M. , Maggi, S. , Bertocco, A. , Bano, G. , Trevisan, C. , Manzato, E. , Sergi, G. , Schofield, P. , Kouidrat, Y. , Veronese, N. , & Stubbs, B. (2017). Low vitamin D levels increase the risk of type 2 diabetes in older adults: A systematic review and meta‐analysis. Maturitas, 100, 8–15. 10.1016/j.maturitas.2017.02.016 28539181

[fsn32488-bib-0044] Lugon‐Moulin, N. , Martin, F. , Krauss, M. R. , Ramey, P. B. , & Rossi, L. (2006). Cadmium concentration in tobacco (*Nicotiana tabacum* L.) from different countries and its relationship with other elements. Chemosphere, 63, 1074–1086. 10.1016/j.chemosphere.2005.09.005 16310829

[fsn32488-bib-0045] Ma Moun, L. , Simar, D. , Malatesta, D. , Caillaud, C. , Peruchon, E. , Couret, I. , Rossi, M. , & Mariano‐Goulart, D. (2005). Response of bone metabolism related hormones to a single session of strenuous exercise in active elderly subjects. British Journal of Sports Medicine, 39, 497–502. 10.1136/bjsm.2004.013151 16046330 PMC1725278

[fsn32488-bib-0046] MacLaughlin, J. A. , Anderson, R. R. , & Holick, M. F. (1982). Spectral character of sunlight modulates photosynthesis of previtamin D3 and its photoisomers in human skin. Science, 216, 1001–1003. 10.1126/science.6281884 6281884

[fsn32488-bib-0047] Matsunawa, M. , Amano, Y. , Endo, K. , Uno, S. , Sakaki, T. , Yamada, S. , & Makishima, M. (2009). The aryl hydrocarbon receptor activator benzo[a]pyrene enhances vitamin D3 catabolism in macrophages. Toxicological Sciences, 109, 50–58. 10.1093/toxsci/kfp044 19244278

[fsn32488-bib-0048] McCulloch, R. G. , Whiting, S. J. , Bailey, D. A. , & Houston, C. S. (1991). The effect of cigarette smoking on trabecular bone density in premenopausal women, aged 20–35 years. Canadian Journal of Public Health, 82, 434–435. 1790511

[fsn32488-bib-0049] Morabia, A. , Bernstein, M. S. , & Antonini, S. (2000). Smoking, dietary calcium and vitamin D deficiency in women: A population‐based study. European Journal of Clinical Nutrition European Journal of Clinical Nutrition, 54, 684–689. 10.1038/sj.ejcn.1601074 11002379

[fsn32488-bib-0050] Mulligan, J. K. , Nagel, W. , O'Connell, B. P. , Wentzel, J. , Atkinson, C. , & Schlosser, R. J. (2014). Cigarette smoke exposure is associated with vitamin D3 deficiencies in patients with chronic rhinosinusitis. Journal of Allergy and Clinical Immunology, 134, 342–349. 10.1016/j.jaci.2014.01.039 24698317

[fsn32488-bib-0051] Need, A. G. , Kemp, A. , Giles, N. , Morris, H. A. , Horowitz, M. , & Nordin, B. E. C. (2002). Relationships between intestinal calcium absorption, serum vitamin D metabolites and smoking in postmenopausal women. Osteoporosis International, 13, 83–88. 10.1007/s198-002-8342-9 11883410

[fsn32488-bib-0052] Okan, F. , Okan, S. , & Zincir, H. (2020). Effect of sunlight exposure on vitamin D status of individuals living in a nursing home and their own homes. Journal of Clinical Densitometry, 23, 21–28. 10.1016/j.jocd.2018.12.005 30655188

[fsn32488-bib-0053] Omdahl, J. L. , Morris, H. A. , & May, B. K. (2002). Hydroxylase enzymes of the vitamin D pathway: Expression, function, and regulation. Annual Review of Nutrition, 22, 139–166. 10.1146/annurev.nutr.22.120501.150216 12055341

[fsn32488-bib-0054] Ortego‐Centeno, N. , Mu Oz‐Torres, M. , Jódar, E. , Hernández‐Quero, J. , Jurado‐Duce, A. , & de la Higuera Torres‐Puchol, J. (1997). Effect of tobacco consumption on bone mineral density in healthy young males. Calcified Tissue International, 60, 496–500. 10.1007/s002239900270 9164822

[fsn32488-bib-0055] Paik, J. M. , Curhan, G. C. , Forman, J. P. , & Taylor, E. N. (2010). Determinants of plasma parathyroid hormone levels in young women. Calcified Tissue International, 87, 211–217. 10.1007/s00223-010-9397-5 20631996 PMC3079245

[fsn32488-bib-0056] Pillai, S. , Oresajo, C. , & Hayward, J. (2005). Ultraviolet radiation and skin aging: Roles of reactive oxygen species, inflammation and protease activation, and strategies for prevention of inflammation‐induced matrix degradation ‐ A review. International Journal of Cosmetic Science, 27, 17–34. 10.1111/j.1467-2494.2004.00241.x 18492178

[fsn32488-bib-0057] Rapuri, P. B. , Gallagher, J. C. , Balhorn, K. E. , & Ryschon, K. L. (2000). Smoking and bone metabolism in elderly women. Bone, 27, 429–436. 10.1016/S8756-3282(00)00341-0 10962356

[fsn32488-bib-0058] Redington, K. (1984). Taste differences between cigarette smokers and nonsmokers. Pharmacology Biochemistry and Behavior, 21, 203–208. 10.1016/0091-3057(84)90215-6 6483932

[fsn32488-bib-0059] Richard, A. , Rohrmann, S. , & Quack Lötscher, K. (2017). Prevalence of vitamin D deficiency and its associations with skin color in pregnant women in the first trimester in a sample from Switzerland. Nutrients, 9, 260. 10.3390/nu9030260 PMC5372923 28287422

[fsn32488-bib-0060] Schierbeck, L. L. , Rejnmark, L. , Tofteng, C. L. , Stilgren, L. , Eiken, P. , Mosekilde, L. , Køber, L. , & Jensen, J.‐E. (2012). Vitamin D deficiency in postmenopausal, healthy women predicts increased cardiovascular events: A 16‐ year follow‐up study. European Journal of Endocrinology, 167, 553–560. 10.1530/EJE-12-0283 22875588

[fsn32488-bib-0061] Schmitt, E. B. , Nahas‐Neto, J. , Bueloni‐Dias, F. , Poloni, P. F. , Orsatti, C. L. , & Petri Nahas, E. A. (2018). Vitamin D deficiency is associated with metabolic syndrome in postmenopausal women. Maturitas, 107, 97–102. 10.1016/j.maturitas.2017.10.011 29169589

[fsn32488-bib-0062] Schwarz, P. , Sørensen, H. A. , & Transbøl, I. (1994). Inter‐relations between the calcium set‐points of Parfitt and Brown in primary hyperparathyroidism: A sequential citrate and calcium clamp study. European Journal of Clinical Investigation, 24, 553–558. 10.1111/j.1365-2362.1994.tb01106.x 7982443

[fsn32488-bib-0063] Scragg, R. , Holdaway, I. , Jackson, R. , & Lim, T. (1992). Plasma 25‐hydroxyvitamin D3 and its relation to physical activity and other heart disease risk factor s in the general population. Annals of Epidemiology, 2, 697–703. 10.1016/1047-2797(92)90014-H 1342321

[fsn32488-bib-0064] Scragg, R. , Holdaway, I. , Singh, V. , Metcalf, P. , Baker, J. , & Dryson, E. (1995). Serum 25‐hydroxyvitamin D3 is related to physical activity and ethnicity but not obesity in a multicultural workforce. Australian and New Zealand Journal of Medicine, 25, 218–223. 10.1111/j.1445-5994.1995.tb01526.x 7487689

[fsn32488-bib-0065] Shen, G. S. , Li, Y. , Zhao, G. Y. , Zhou, H. B. , Xie, Z. G. , Xu, W. , Chen, H. N. , Dong, Q. R. , & Xu, Y. J. (2015). Cigarette smoking and risk of hip fracture in women: A meta‐analysis of prospective cohort studies. Injury, 46, 1333–1340. 10.1016/j.injury.2015.04.008 25956674

[fsn32488-bib-0066] Smith, C. J. , & Hansch, C. (2000). The relative toxicity of compounds in mainstream cigarette smoke condensate. Food and Chemical Toxicology, 38, 637–646. 10.1016/S0278-6915(00)00051-X 10942325

[fsn32488-bib-0067] Stürmer, M. , Ebeková, K. , Fazeli, G. , Bahner, U. , Stäb, F. , & Heidland, A. (2015). 25‐hydroxyvitamin d and advanced glycation endproducts in healthy and hypertensive subjects: Are there interactions? Journal of Renal Nutrition, 25, 209–216. 10.1053/j.jrn.2014.10.027 25600393

[fsn32488-bib-0068] Supervía, A. , Nogués, X. , Enjuanes, A. , Vila, J. , Mellibovsky, L. , Serrano, S. , Aubía, J. , & Díez‐Pérez, A. (2006). Effect of smoking and smoking cessation on bone mass, bone remodeling, vitamin D, PTH and sex hormones. Journal of Musculoskeletal and Neuronal Interactions, 6, 234–241. 17142943

[fsn32488-bib-0069] Thuesen, B. , Husemoen, L. , Fenger, M. , Jakobsen, J. , Schwarz, P. , Toft, U. , Ovesen, L. , Jørgensen, T. , & Linneberg, A. (2012). Determinants of vitamin D status in a general population of Danish adults. Bone, 50, 605–610. 10.1016/j.bone.2011.12.016 22227435

[fsn32488-bib-0070] Tonnesen, R. , Hovind, P. H. , Jensen, L. T. , & Schwarz, P. (2016). Determinants of vitamin D status in young adults: Influence of lifestyle, sociodemographic and anthropometric factors. BMC Public Health, 16, 385. 10.1186/s12889-016-3042-9 27170258 PMC4863340

[fsn32488-bib-0071] Tsutakawa, S. , Kobayashi, D. , Kusama, M. , Moriya, T. , & Nakahata, N. (2009). Nicotine enhances skin necrosis and expression of inflammatory mediators in a rat pressure ulcer model. British Journal of Dermatology, 161, 1020–1027. 10.1111/j.1365-2133.2009.09349.x 19673871

[fsn32488-bib-0072] Umar, M. , Sastry, K. S. , & Chouchane, A. I. (2018). Role of vitamin D beyond the skeletal function: A review of the molecular and clinical studies. International Journal of Molecular Sciences, 19(6), 1618. 10.3390/ijms19061618 PMC6032242 29849001

[fsn32488-bib-0073] Välimäki, M. J. , Kärkkäinen, M. , Lamberg‐Allardt, C. , Laitinen, K. , Alhava, E. , Heikkinen, J. , Impivaara, O. , Mäkelä, P. , Palmgren, J. , Seppänen, R. , Vuori, I. , & Cardiovascular Risk in Young Finns Study Group . (1994). Exercise, smoking, and calcium intake during adolescence and early adulthood as determinants of peak bone mass. BMJ, 309, 230–235. 8069139 10.1136/bmj.309.6949.230 PMC2540782

[fsn32488-bib-0074] Vanherwegen, A. S. , Gysemans, C. , & Mathieu, C. (2017). Vitamin D endocrinology on the cross‐road between immunity and metabolism. Molecular and Cellular Endocrinology, 453, 52–67. 10.1016/j.mce.2017.04.018 28461074

[fsn32488-bib-0075] Ward, K. D. , & Klesges, R. C. (2001). A meta‐analysis of the effects of cigarette smoking on bone mineral density. Calcified Tissue International, 68, 259–270. 10.1007/BF02390832 11683532 PMC5352985

[fsn32488-bib-0076] Xu, H. W. , Yi, Y. Y. , Zhang, S. B. , Hu, T. , Wang, S.‐J. , Zhao, W.‐D. , & Wu, D.‐S. (2020). Does vitamin D status influence lumbar disc degeneration and low back pain in postmenopausal women? A retrospective single‐center study. Menopause, 27, 586–592. 10.1097/GME.0000000000001499 32049928

[fsn32488-bib-0077] Zhou, R. , Wang, M. , Huang, H. , Li, W. , Hu, Y. , & Wu, T. (2018). Lower vitamin D status is associated with an increased risk of ischemic stroke: A systematic review and meta‐analysis. Nutrients, 10(3), 277. 10.3390/nu10030277 PMC5872695 29495586

